# Supplementary material for: BPG4 regulates chloroplast development and homeostasis by suppressing GLK transcription factors and involving light and brassinosteroid signaling
Source: Nat Commun. 2024 Jan 8;15:370. doi: 10.1038/s41467-023-44492-5 (PMC10774444; doi:10.1038/s41467-023-44492-5)
Supplement: Supplementary file 1 — Supplementary Information [file 41467_2023_44492_MOESM1_ESM.pdf]

BPG4 regulates chloroplast development and homeostasis by suppressing GLK transcription factors by involving light and brassinosteroid signaling.

Ryo Tachibana[1], Susumu Abe[2, 3], Momo Marugami[2, 3], Ayumi Yamagami[1], Rino Akema[1], Takao Ohashi[1], Kaisei Nishida[1], Shohei Nosaki[4], Takuya Miyakawa[1], Masaru Tanokura[5], Jong-Myong Kim[3, 5, 6], Motoaki Seki[3], Takehito Inaba[7], Minami Matsui[3], Kentaro Ifuku[8], Tetsuo Kushiro[2], Tadao Asami[5], Takeshi Nakano[1*]

Supplementary materials
Supplementary Figure 1. Even in the presence of Brz, *BPG4-OX* transgenic plants possessed the pale-green leaves identical to those of *bpg4-1D*
Supplementary Figure 2. Location of T-DNA insertions in *bpg4-1* and *bpg4-2*, mutations by the CRISPR/Cas9 system in *bgh1-1*, *bgh2-1*, *bgh2-2*, *bgh3-1*, and *bgh3-2*, and relative expression levels of *BGH2* and *BGH3* in *BGH2/3* overexpressing plants.
Supplementary Figure 3. Adults phenotypes of WT, *bpg4-1*, *bpg4-2*, *BPG4-OX-2*, and *BPG4-OX-10* grown in soil.
Supplementary Figure 4. Sequence alignment and mRNA expression pattern of Arabidopsis BPG4 family.
Supplementary Figure 5. Phenotypes of *BGH* knockout and overexpressing plants, and double knockout plants of *BPG4* and *BGHs*.
Supplementary Figure 6. Immunofluorescence staining for the detection of endogenous BPG4 protein.
Supplementary Figure 7. Relative expression levels of genes encoding chlorophyll-related enzymes and photosynthesis-associated genes in WT, *bpg4-1*, *bpg4-2*, *BPG4-OX-2*, and *BPG4-OX-10*.
Supplementary Figure 8. Y2H assays to test the interactions of BPG4 with photosynthesis-associated factors.
Supplementary Figure 9. Negative controls and confirmation of *nYFP* and *cYFP* expression in suspension-cultured cells, and negative control of Y2H assay.
Supplementary Figure 10. Probe sequences for EMSAs to analyze GLK1 DNA-binding ability.
Supplementary Figure 11. Phenotypes and *PhANG* expression of *bpg4-1GLK1-OX*.
Supplementary Figure 12. *BPG4* expression was induced in BR-deficient mutants and BR-signaling mutants.
Supplementary Figure 13. Probe sequences for EMSAs and EMSA results depending on the amount of MBP-BES1 DBD.
Supplementary Figure 14. BPG4 protein status and accumulation were not altered by λPP and bikinin treatment.
Supplementary Figure 15. BPG4 protein status and accumulation in BL and Brz treatment, and BR-signaling mutants.
Supplementary Figure 16. *BPG4* and photosynthesis-associated gene expression in response to light exposure

Supplementary table S1-S10. The list of primers used in this study.

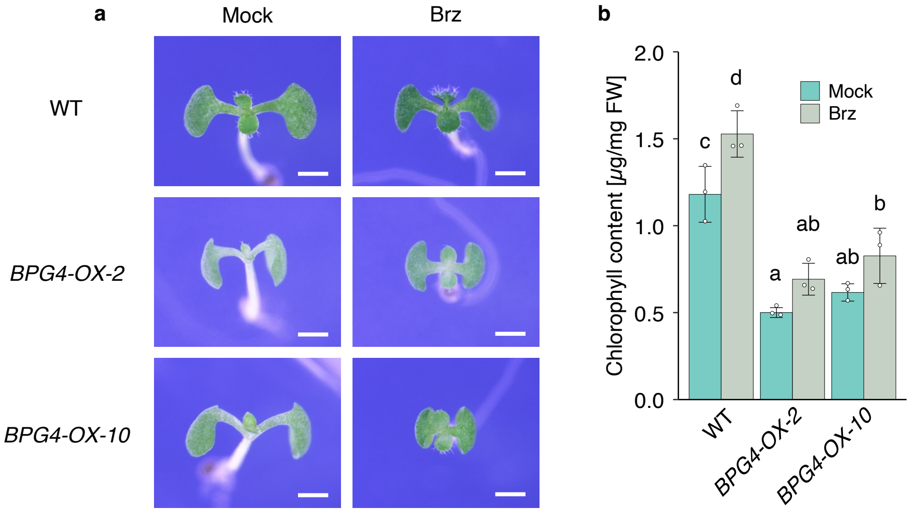

Supplementary Figure 1. Even in the presence of Brz, *BPG4-OX* transgenic plants possessed the pale-green leaves identical to those of *bpg4-1D*.

A, WT (Col-0), *BPG4-OX-2*, and *BPG4-OX-10* seedlings grown on half-strength MS medium supplemented with 1 μM Brz or the same volume of DMSO solvent (mock) for 8 days. Scale bars = 1 mm.

b, Endogenous contents of total chlorophyll in the WT, *BPG4-OX-2*, and *BPG4-OX-10* plants shown in Supplementary Figure 1a. The means and SDs were obtained from 14-16 plants, with three biological replicates.

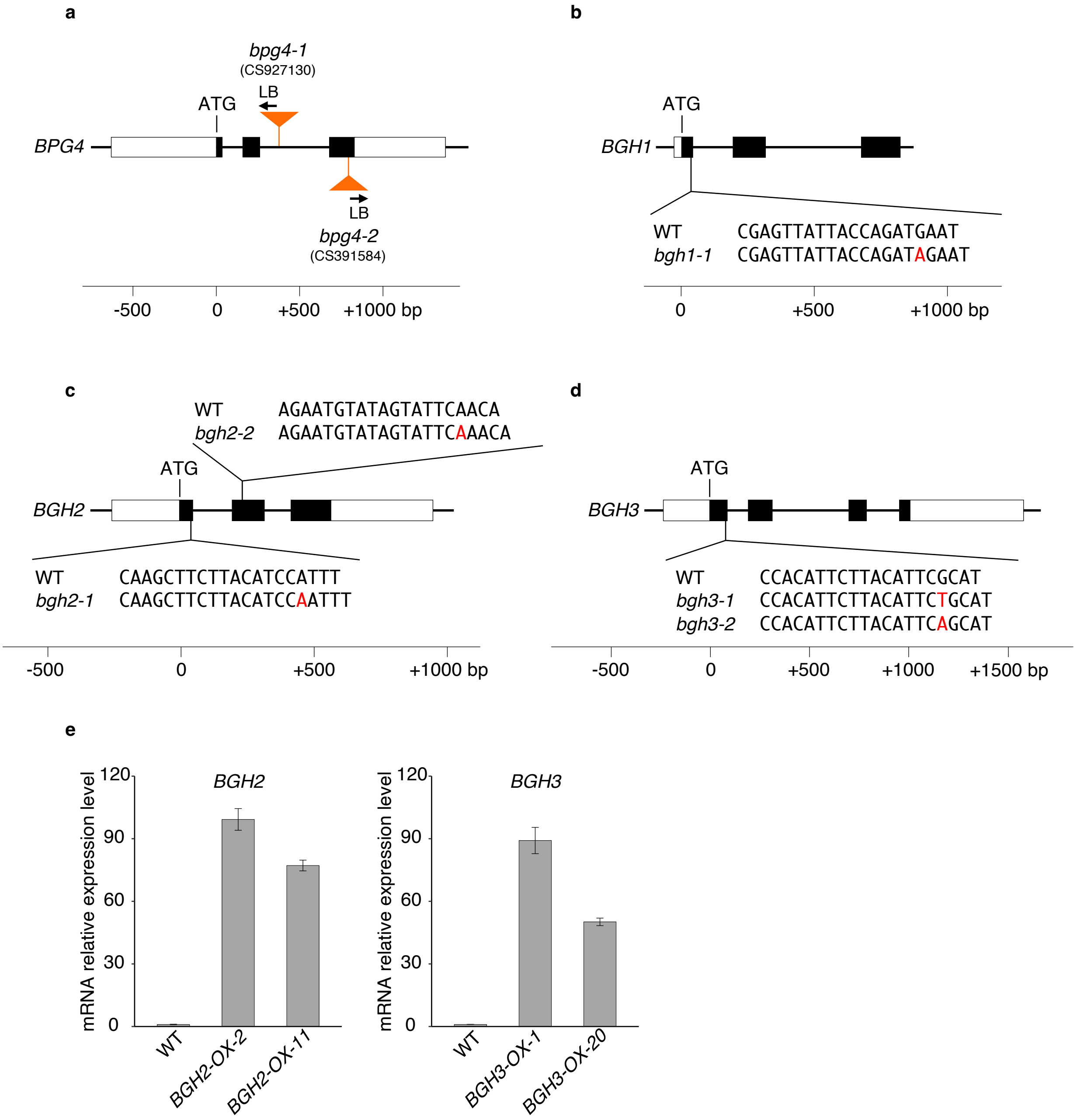

Supplementary Figure 2. Location of T-DNA insertions in *bpg4-1* and *bpg4-2*, mutations by the CRISPR/Cas9 system in *bgh1-1*, *bgh2-1*, *bgh2-2*, *bgh3-1*, and *bgh3-2*, and relative expression levels of *BGH2* and *BGH3* in *BGH2/3* overexpressing plants.

a, Gene structure of *BPG4* indicating T-DNA insertions that cause mutations. The T-DNA causing the *bpg4-1* mutation was inserted 380 bp downstream of the start codon (ATG). The T-DNA causing the *bpg4-2* mutation was inserted 806 bp downstream of the start codon (ATG). The black box indicates an exon region, and the white boxes indicate untranslated regions (UTRs).

b, c, d, Gene structure of *BGH1* (b), *BGH2* (c), and *BGH3* (d) indicating mutations by the CRISPR/Cas9 system.

e, Relative expression of *BGH2* in WT (Col-0), *BGH2-OX-2*, and *BGH2-OX-11* and *BGH3* in WT, *BGH3-OX-1*, and *BGH3-OX-20* grown on half-strength MS medium for 7 days. The relative expression levels were normalized to GAPDH. Data are presented as the means +/– SDs of at least three technical replicates.

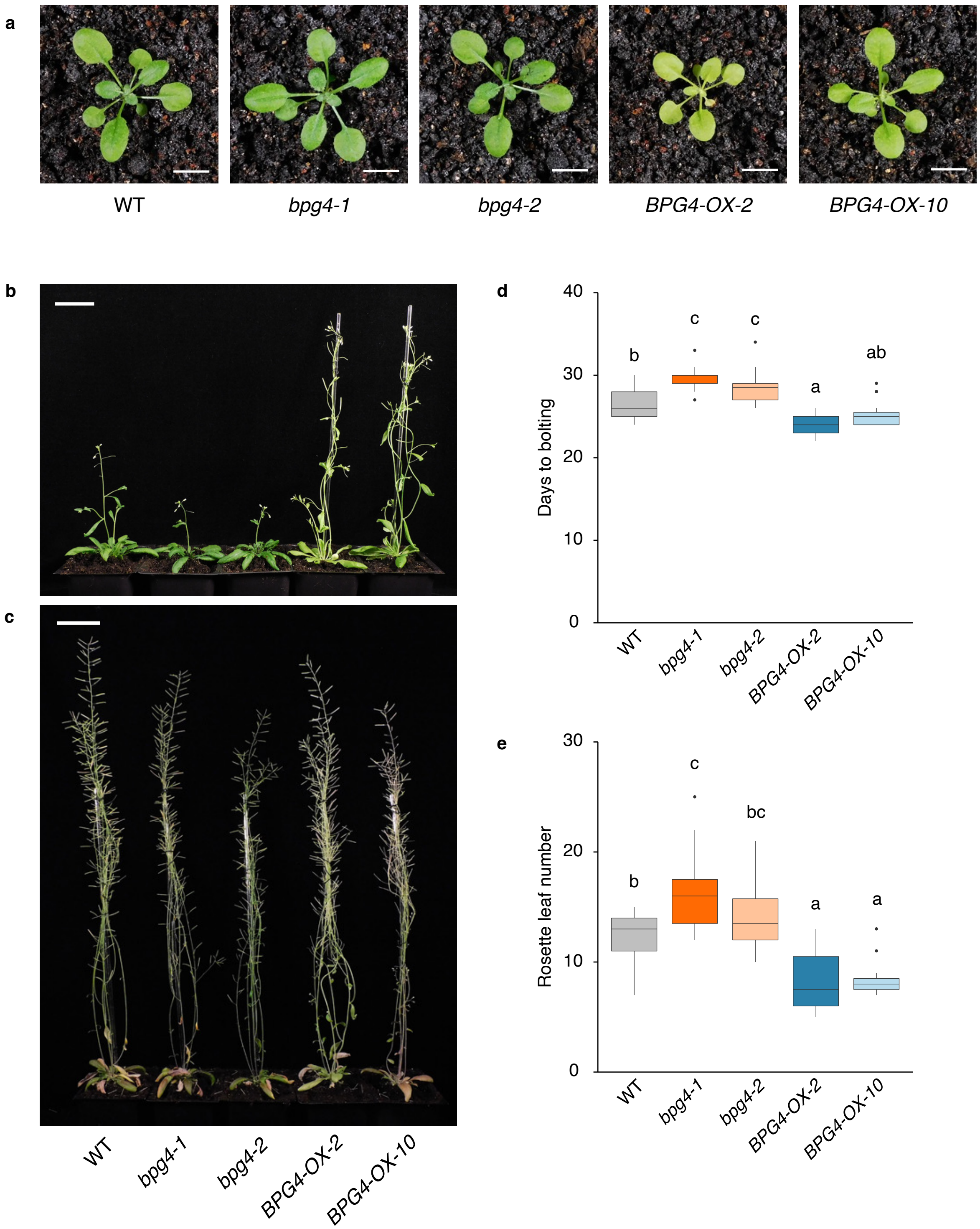

Supplementary Figure 3. Adults phenotypes of WT, *bpg4-1*, *bpg4-2*, *BPG4-OX-2*, and *BPG4-OX-10* grown in soil.

a, WT (Col-0), *bpg4-1*, *bpg4-2*, *BPG4-OX-2*, and *BPG4-OX-10* plants grown in soil for 3 weeks under weak light (60 μmol photons m^{-2} s^{-1}). Scale bars = 1 cm.

b, c, WT, *bpg4-1*, *bpg4-2*, *BPG4-OX-2*, and *BPG4-OX-10* plants grown in soil for 5 weeks (b) and 8 weeks (c) under moderately strong light (150 μmol photons m^{-2} s^{-1}). Scale bars = 5 cm.

d, e, The number of days from sowing to the days when the first inflorescence shoot had elongated (d), and the number of rosette leaves when the first flower had opened (e) in WT, *bpg4-1*, *bpg4-2*, *BPG4-OX-2*, and *BPG4-OX-10* grown in soil under growth light (100 μmol photons m^{-2} s^{-1}). $n \geq 13$ biological samples. The center of the boxplot is denoted by the median, a horizontal line dividing the box into two equal halves. The bounds of the box are defined by the lower quartile (25th percentile) and the upper quartile (75th percentile). The whiskers extend from the box and represent the data points that fall within 1.5 times the IQR from the lower and upper quartiles. Any data point outside this range is considered an outlier and plotted individually.

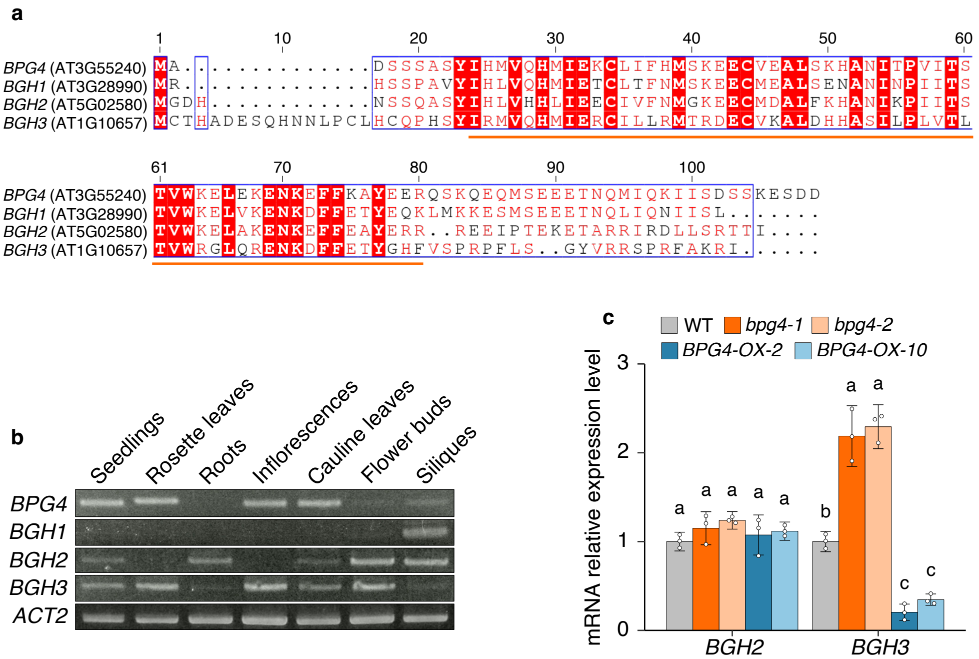

Supplementary Figure 4. Sequence alignment and mRNA expression pattern of the Arabidopsis BPG4 family.

a, Sequence alignment of BPG4 and BGHs in Arabidopsis. The bars with orange under the sequence indicate the A_thal_3526 domain.

b, RT–PCR analysis of *BPG4* and *BGHs* expression in various tissues in WT (Col-0). Plants were grown on half-strength MS medium for 8 days (the whole tissues of seedlings), soil for 24 days (rosette leaves and roots) or soil for 42 days (inflorescences, cauline leaves, flower buds, and siliques). *ACT2* was used as the control.

c, Relative expression of *BGH2* and *BGH3* in WT, *bpg4-1*, *bpg4-2*, *BPG4-OX-2*, and *BPG4-OX-10*. Plants were grown under the same conditions as were those in Figure 2b. The relative expression levels were normalized to *GAPDH*. The means and SDs were obtained from three biological replicates. The different letters above the bars indicate statistically significant differences between the samples.

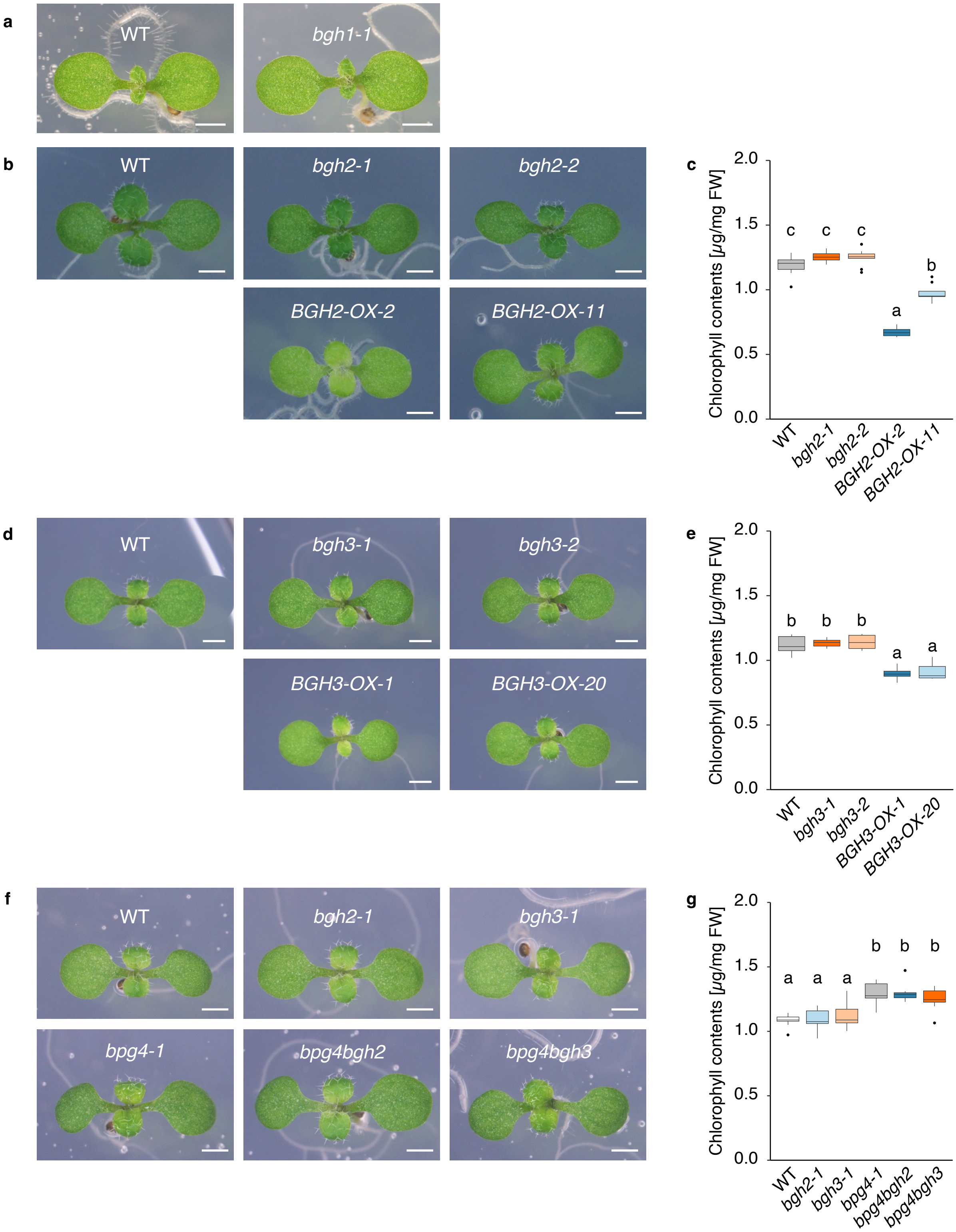

Supplementary Figure 5. Phenotypes of *BGH* knockout and overexpressing plants, and double knockout plants of *BPG4* and *BGHs*.

a, WT (Col-0), and *bgh1-1* seedlings grown on half-strength MS medium for 7 days. Scale bars = 1 mm. b–g, Phenotypes (b, d, f) and endogenous contents of chlorophyll (c, e, g) in WT, *bgh2-1*, *bgh2-2*, *BGH2-OX-2* and *BGH2-OX-11* seedlings (b, c), WT, *bgh2-1*, *bgh2-2*, *BGH3-OX-1* and *BGH3-OX-20* seedlings (d, e), and WT, *bpg4-1*, *bgh2-1*, *bgh3-1*, *bpg4bgh2*, and *bpg4bgh3* seedlings (f, g) grown on half-strength MS medium for 8 days. Scale bars = 1 mm. The boxplot was obtained from 30 mg plant tissue and nine biological replicates. The different letters above the bars indicate statistically significant differences between the samples.

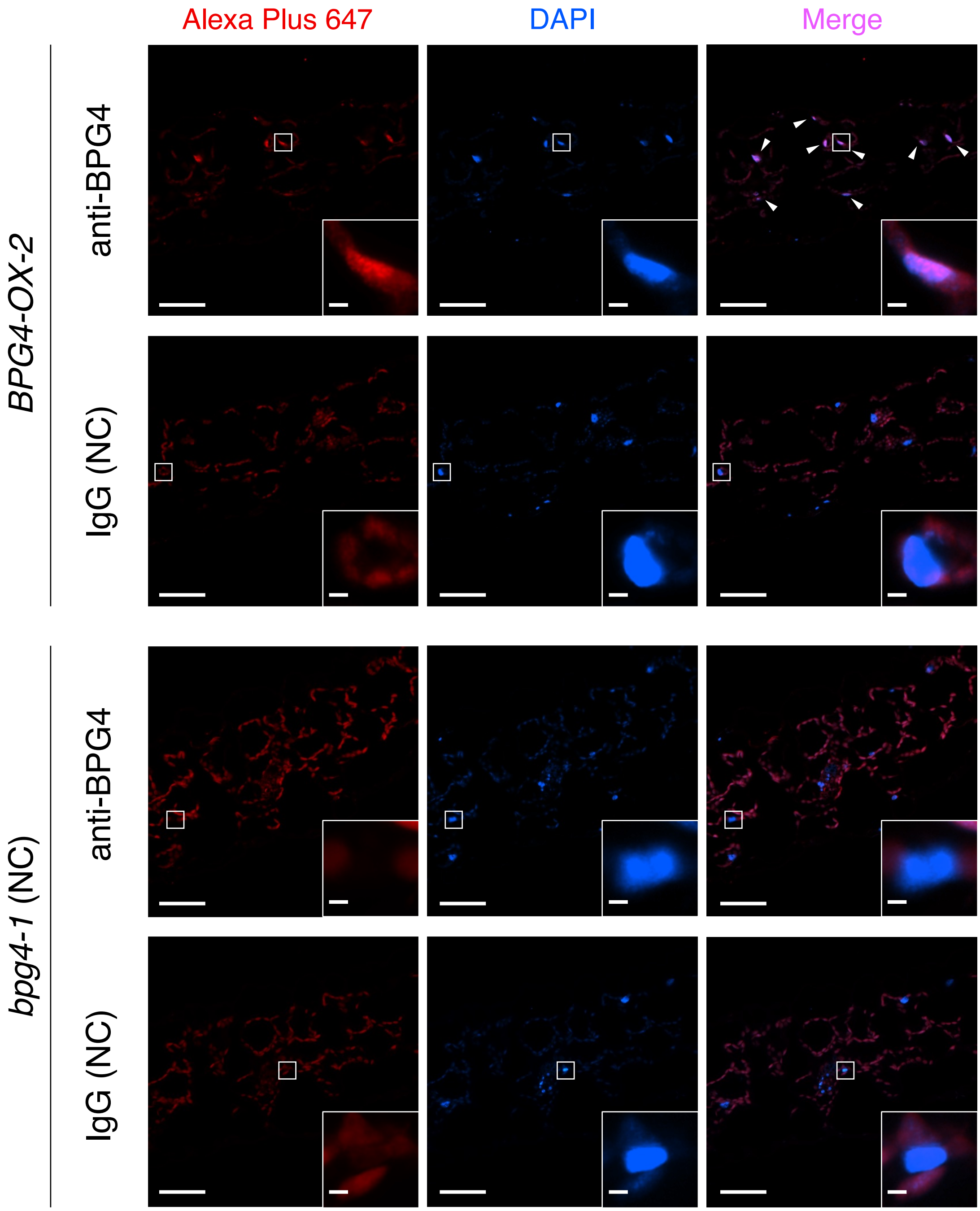

Supplementary Figure 6. Immunofluorescence staining for the detection of endogenous BPG4 protein.

Subcellular localization patterns of endogenous BPG4 proteins in cotyledon epidermal cells of *BPG4-OX-2*, and *bpg4-1* seedlings grown on half-strength MS medium for 9 days. Endogenous BPG4 proteins were stained with anti-BPG4 antibodies or normal rabbit IgG, followed by donkey anti-rabbit IgG secondary antibody conjugated with Alexa Fluor Plus 647 (red). Normal rabbit IgG and *bpg4-1* were used as negative controls (NCs) to demonstrate nonspecific signals or autofluorescence of chloroplasts. Nuclei were stained with DAPI (blue). Inset panels show enlarged views of the representative nuclei, which are surrounded by a white square in each panel. White arrowheads exhibit the fluorescent signals by anti-BPG4 antibodies overlapping with the DAPI signals. Scale bars = 50 μm and 2.5 μm (inset).

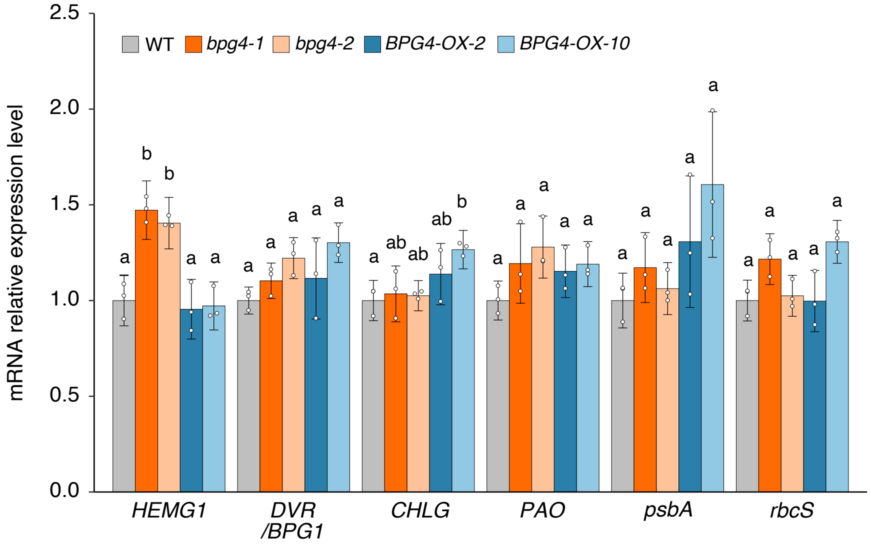

Supplementary Figure 7. Relative expression levels of genes encoding chlorophyll-related enzymes and photosynthesis-associated genes in WT, *bpg4-1*, *bpg4-2*, *BPG4-OX-2*, and *BPG4-OX-10*.

Plants were grown under the same conditions as were those in Figure 3c. The genes include Pheide *a* oxygenase (*PAO*); PSII D1 protein (*psbA*); and Rubisco small subunit (*rbcS*). The relative expression levels were normalized to *GAPDH*. The means and SDs were obtained from three biological replicates. The different letters above the bars indicate statistically significant differences between the samples.

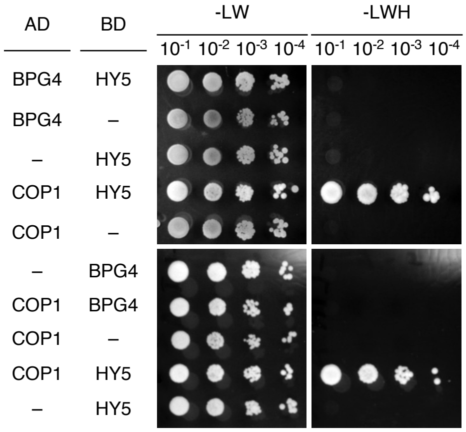

Supplementary Figure 8. Y2H assays to test the interactions of BPG4 with photosynthesis-associated factors.

The interactions between COP1-HY5 were used as positive controls.

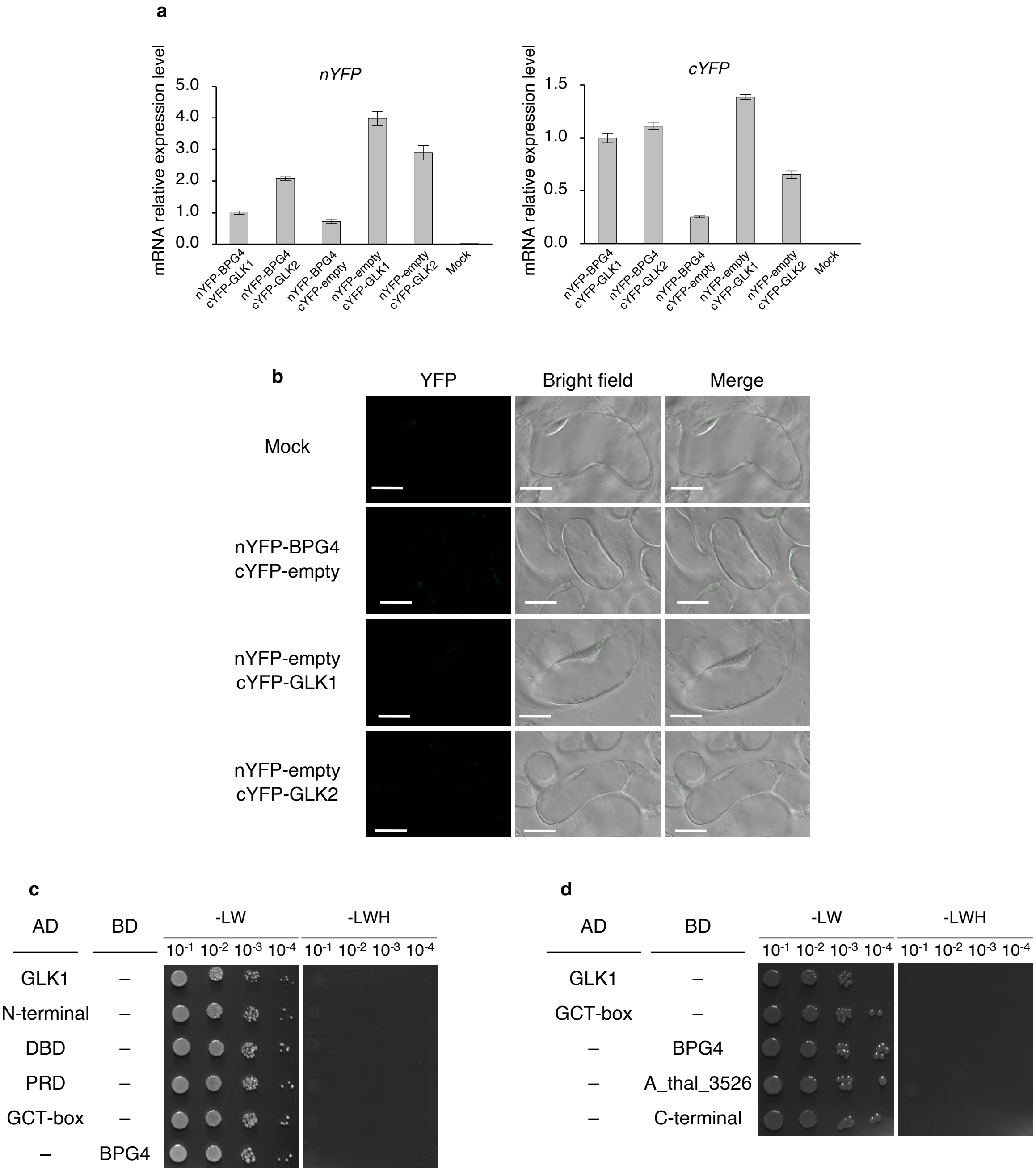

Supplementary Figure 9. Negative controls and confirmation of *nYFP* and *cYFP* expression in suspension-cultured cells, and negative controls of the Y2H assay.

a, Relative expression of *nYFP* and *cYFP* in suspension-cultured cells. The relative expression levels were normalized to *ACT2* and represented as *n*-fold changes relative to the value of nYFP-BPG4 cYFP-GLK1. Data are presented as the means +/– SDs of at least three technical replicates.

b, Negative controls of BiFC assays of the interactions of BPG4 with GLK1 and GLK2.

c, d, Negative controls of the Y2H assays in Figure 4f (c) and 4g (d).

CAO-probe	TTTAGATCCCAT**GATTCT**CAACACGACTGA

Supplementary Figure 10. Probe sequences for EMSAs to analyze GLK1 DNA-binding ability. Sequences of probes used. The sequences with a bar denotes a GLK-binding site.

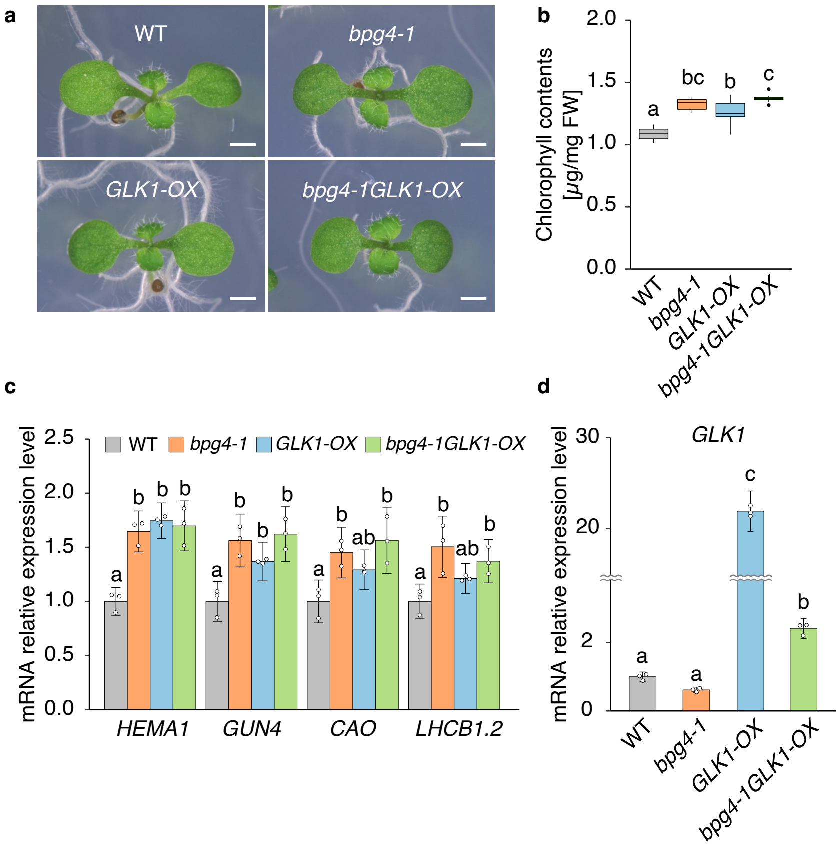

Supplementary Figure 11. Phenotypes and *PhANG* expression of *bpg4-1GLK1-OX*.

a, b, Phenotypes (a) and endogenous contents of total chlorophylls (b) in WT (Col-0), *bpg4-1*, *GLK1-OX*, and *bpg4-1 GLK1-OX* seedlings grown on half-strength MS medium for 8 days. Scale bars = 1 mm; The boxplot was obtained from 30 mg plant tissue and nine biological replicates. The different letters above the bars indicate statistically significant differences between the samples.

c, d, Relative expression of *PhANGs* (c) and *GLK1* (d) in WT, *bpg4-1*, *GLK1-OX*, and *bpg4-1 GLK1-OX* seedlings grown on half-strength MS medium for 8 days. The relative expression levels were normalized to GAPDH. The means and SDs were obtained from three biological replicates. The different letters above the bars indicate statistically significant differences between the samples.

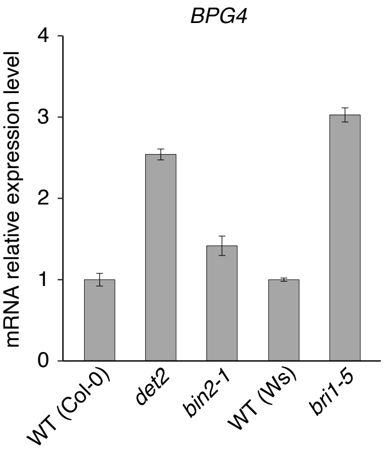

Supplementary Figure 12. *BPG4* expression was induced in BR-deficient mutants and BR-signaling mutants.

Relative expression of *BPG4* in WT (Col-0), *det2*, *bin2-1*, WT (Ws) and *bri1-5* seedlings grown on half-strength MS medium for 8 days. The relative expression levels were normalized to *GAPDH*. Data are presented as the means +/– SDs of at least three technical replicates.

a

BPG4 G-box-1	GGGGCCATGGAT**CACGTG**GAGGATGCGTGA
BPG4 G-box-2	TGATCAATCTCC**CACGTG**GGGCTTCTGGCC
BPG4 mG-box-1	GGGGCCATGGAT**AAAAAA**GAGGATGCGTGA
BPG4 mG-box-2	TGATCAATCTCC**AAAAAA**GGGCTTCTGGCC

b

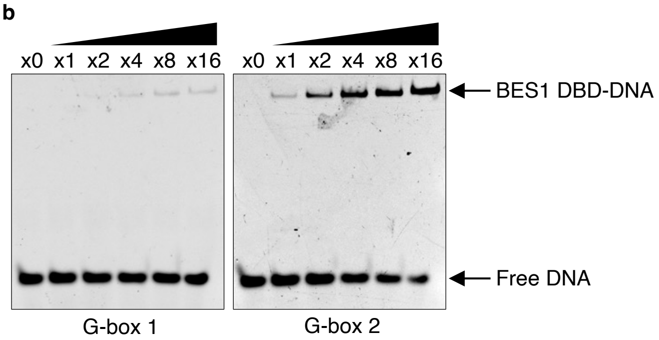

Supplementary Figure 13. Probe sequences for EMSAs and EMSA results depending on the amount of MBP-BES1 DBD.

a, List of sequences of probes used. The mutant probe is the probe with a mutation in which CACGTG was changed to AAAAAA.

b, EMSA results of MBP-fused BES1 DBD with a FAM-labeled ds *BPG4* promoter DNA probe containing a G-box (CACGTG). The molar ratios of protein-to-DNA are shown at the top of each gel.

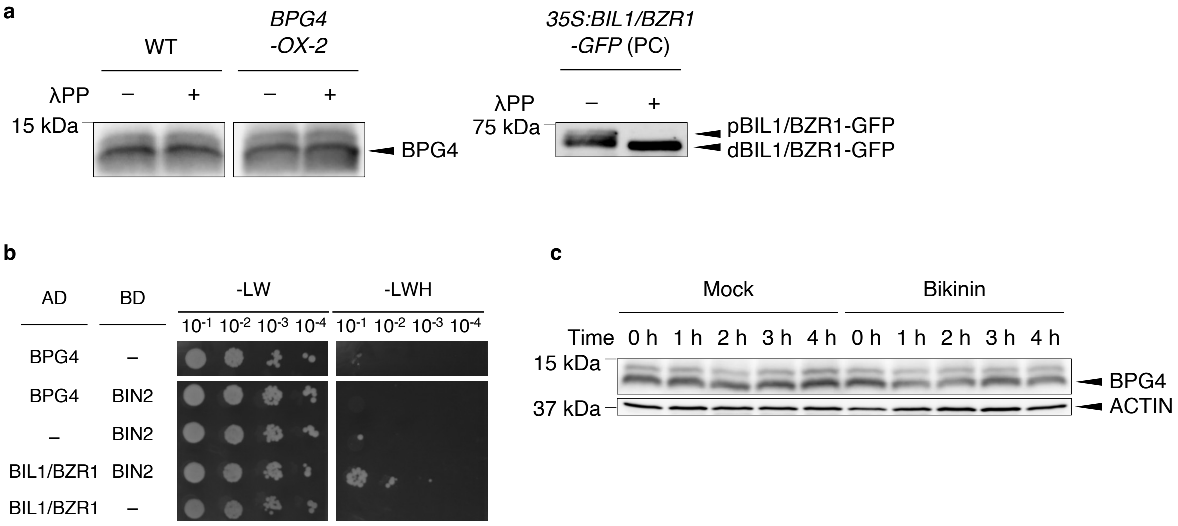

Supplementary Figure 14. BPG4 protein status and accumulation were not altered by λPP and bikinin treatment.

a, Detection of BPG4 proteins using an anti-BPG4 antibody in WT (Col-0) and *BPG4-OX-2* seedlings treated with lambda protein phosphatase (λPP) after being grown on half-strength MS medium for 8 days. BIL1/BZR1-GFP was used as a positive control (PC) for λPP treatment. pBIL1/BZR1-GFP, phosphorylated BIL1/BZR1-GFP; dBIL1/BZR1-GFP, dephosphorylated BIL1-BZR1-GFP.

b, Y2H assays to test the interactions of BPG4 with BIN2. The interaction between BIN2-BIL1/BZR1 was used as a positive control.

c, Detection of BPG4 proteins using an anti-BPG4 antibody in *BPG4-OX-2* seedlings treated with 50 μM bikinin or the same volume of DMSO solvent (mock) for 0, 1, 2, 3, or 4 h after being grown on half-strength MS medium for 11 days. ACTIN was used as a loading control.

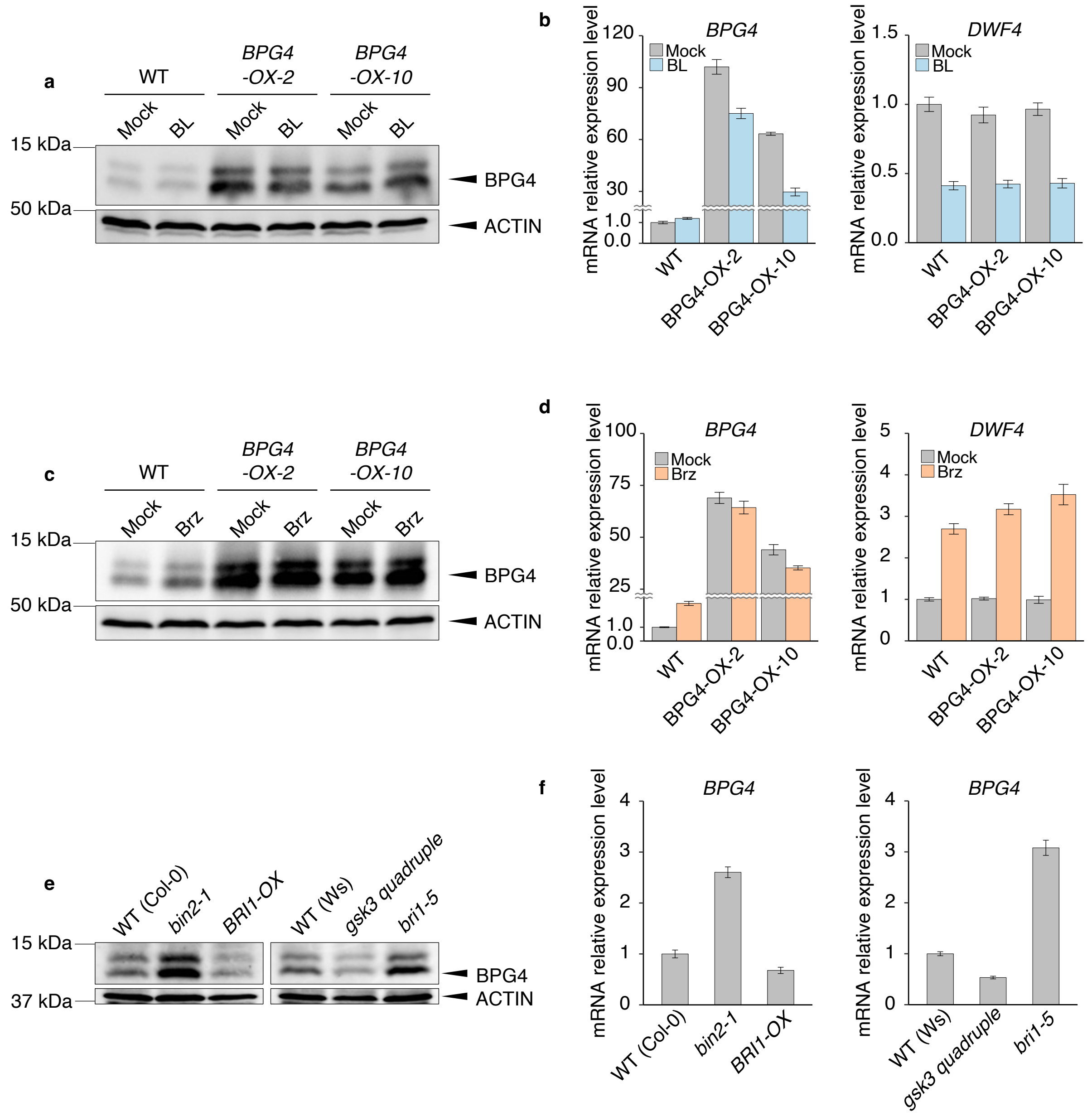

Supplementary Figure 15. BPG4 protein status and accumulation in BL and Brz treatment, and BR-signaling mutants.

a, b, Detection of BPG4 proteins using an anti-BPG4 antibody (a) and relative expression of *BPG4* (b) in WT (Col-0), *BPG4-OX-2*, and *BPG4-OX-10* seedlings grown on half-strength MS medium supplemented with 100 nM BL or the same volume of DMSO solvent (mock) for 8 days. ACTIN was used as a loading control. The relative expression levels were normalized to *GAPDH*. Data are presented as the means +/– SDs of at least three technical replicates. *DWF4* was used as a positive control for BL treatment.

c, d, Detection of BPG4 proteins using an anti-BPG4 antibody (c) and relative expression of *BPG4* (d) in WT, *BPG4-OX-2*, and *BPG4-OX-10* seedlings grown on half-strength MS medium supplemented with 3 μM Brz or the same volume of DMSO solvent (mock) for 9 days. Actin was used as a loading control. The relative expression levels were normalized to *GAPDH*. Data are presented as the means +/– SDs of at least three technical replicates. *DWF4* was used as a positive control for Brz treatment.

e, Detection of BPG4 proteins using an anti-BPG4 antibody (e) and relative expression of *BPG4* (f) in WT (Col-0), *bin2-1*, *BRI1-OX*, WT (Ws), *gsk3 quadruple* (*bin2 bil1 bil2 ATSK13RNAi*), and *bri1-5* seedlings grown on half-strength MS medium for 7 days. ACTIN was used as a loading control. The relative expression levels were normalized to *GAPDH*. Data are presented as the means +/– SDs of at least three technical replicates.

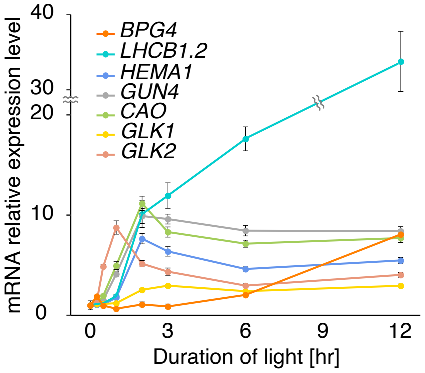

Supplementary Figure 16. *BPG4* and photosynthesis-associated gene expression in response to light exposure

Enlarged view of Figure 7d.

Supplementary Table S1: Primers list for screening of the bpg4-1D mutant

Primer name	Sequence (5' - 3')
FOX−F3	GTACGTATTTTTACAACAATTACCAACAAC
FOX−R3	CAAATGTTTGAACGATCGGGGAAAT

Supplementary Table S2: Primers list for generation of transgenic plants

Primer name	Sequence (5' - 3')
BPG4-CDS-F1	CACCATGGCAGATTCTTCTTCTGCT
BPG4-CDS-R1	TCAGTCGTCGGATTCTTTAGA
BPG4-cDNA-no-stop-R1	GTCGTCGGATTCTTTAGATGA
BPG4-promoter-F1	CACCGAGGTCCACATGATGAGGAA
BPG4-first-exon-R1	CATGTGAATGTAAGAAGCAGAA
BGH2-CDS-GW-F1	CACCATGGGTGATCATAATAGCTCG
BGH2-CDS-GW-R1	TTAGATTGTAGTTCGTGAAAGC
BGH3-CDS-GW-F1	CACCATGTGTACTCATGCTGATGAAT
BGH3-CDS-GW-R1	TTATATCCTTTTCGCAAACCTC

Supplementary Table S3: Primers list for generation of mutants

Primer name	Sequence (5' - 3')
BGH1-gRNA1-F	TCCCGCTGTATACATTCATC
BGH1-gRNA2-F	TTCCTCTTTGCTCATGTTAA
BGH2-gRNA1-F	GCAAGCTTCTTACATCCATT
BGH2-gRNA2-F	AGAATGTATAGTATTCAACA
BGH3-gRNA1-F	TGTAAGAATGTGGTTGGCAA
BGH3-gRNA2-F	ACCACATTCTTACATTCGCA
BGH1-gRNAcheck-F	GACCATTCGTCAACATACATAA
BGH1-gRNAcheck-R	AGGACCTATGGATGAAAAATGA
BGH2-gRNAcheck-F	GGGTCTTGTAGCGTTTTATATA
BGH2-gRNAcheck-R	CGTCGGATCATACATATACAC
BGH3-gRNAcheck-F	CTGCGTATGTATATGCATGTG
BGH3-gRNAcheck-R	GCACACTTGCAAAGCCTAATA
BPG4-gRNAcheck-F	ACGATCGAACATCGCGATC
BPG4-gRNAcheck-R	TCAGCCAATTATGATCAATCTC

Supplementary Table S4: Primers list for RT-PCR

Primer name	Sequence (5' - 3')
RT-ACT2-F1	GTGAAGGCTGGATTTGCAGGA
RT-ACT2-R1	AACCACCGATCCAGACACTGT
RT-BGH1-F1	GGTTGTAGCAAAATATATAATAGA
RT-BGH1-R1	CCTTTTTCATAAGCTTCTGCT
RT-BGH2-F1	CCATCATCATATCTCTCTCTA
RT-BGH2-R1	CCCCAAAGGTGAATTATTCATT
RT-BGH3-F1	GAGCGAAGATCAAAGGATAAG
RT-BGH3-R1	GGGAACCTTATGACATCAAAAG
RT-BPG4-F1	ATGGCAGATTCTTCTTCTGCT
RT-BPG4-R1	GTCGTCGGATTCTTTAGATGA

Supplementary Table S5: Primers list for qRT-PCR

Gene	Primer name	Sequence(5' - 3')
GAPDH	qRT-GAPDH-F1	CTGCCCCAAGCAAAGATGCG
	qRT-GAPDH-R1	TTGGCAAGAGGAGCAAGGCA
ACT2	qRT-ACT2-F1	CGCCATCCAAGCTGTTCTC
	qRT-ACT2-R1	TCACGTCCAGCAAGGTCAAG
eIF4a	qRT-eIF4a-F1	TGACCACACAGTCTCTGCAA
	qRT-eIF4a-R1	ACCAGGGAGACTTGTTGGAC
BPG4	qRT-BPG4-F1	GGAATTCTTCAAGGCGTATGA
	qRT-BPG4-R1	CAGTCGTCGGATTCTTTAGATG
HEMA1	qRT-HEMA1-F1	GTGAGCTCTCTGCTTCTTCTGATTCTG
	qRT-HEMA1-R1	CTGCTTCTTTCCTTTGTATATCGATCAGCT
GUN4	qRT-GUN4-F1	GAAACCGCGACCATATTCGAC
	qRT-GUN4-R1	CGGCTTCTCCGGATATCTGAA
CHLH	qRT-CHLH-F1	TGGTAGAGAGACAGAAGCTCGAAA
	qRT-CHLH-R1	CCAAAGAACCTGCCCAAGAG
PORB	qRT-PORB-F1	TGATTACCCTTCAAAGCGTCTCA
	qRT-PORB-R1	CAATGTATTCGTGTTCCCGGT
CAO	qRT-CAO-F1	CCGGTGGAACACGGTTTACTTCTAGATA
	qRT-CAO-R1	AGTATCCTTGGAGACCCGAGGTAGGTGT
LHCB1.2	qRT-LHCB1.2-F1	ATCCGACCGAGTCAAGTACT
	qRT-LHCB1.2-R1	GGTTCCTTGCGAATGTCT
LHCB6	qRT-LHCB6-F1	GGACTTTGAGAAGCTGGAGAGG
	qRT-LHCB6-R1	ACAAACCAAGAGCACCGAGAG
LHCA1	qRT-LHCA1-F1	AAGTACCCGGGAGGCGCATT
	qRT-LHCA1-R1	CGCAAGCCGCCCGTTCT
HEMG1	qRT-HEMG1-F1	TGCCTCCAACACGATCCTT
	qRT-HEMG1-R1	CGGCGGAGGTATTAGTCGTC
DVR/BGH1	qRT-DVR-F1	CGCTTGTAAACCGATCAGCG
	qRT-DVR-R1	AATGGCGTTAATGCCTTGCC
CHLG	qRT-CHLG-F1	TATTATGCGTTGGCGTTGGT
	qRT-CHLG-R1	GCGCTTGCCTGGTACTTGA
PAO	qRT-PAO-F1	GCTCAGACGGCATGGTAAG
	qRT-PAO-R1	GCATCTGACGCTTGGTTAAG
psbA	qRT-psbA-F1	CTATACAACGGCGGTCCTC
	qRT-psbA-R1	GCATACCCAGACGGAAACTA
rbcS	qRT-rbcS-F1	GCACCGACTCCGCTCA
	qRT-rbcS-R1	TGGACTTGACGGGTGTTGTC
GLK1	qRT-GLK1-F1	TTGGGTCTCCGATTCTCCCTAT
	qRT-GLK1-R1	GCAACTGGCGGTGCTCTAAAT
GLK2	qRT-GLK2-F1	TATCCAATGCCGGCCATTGC
	qRT-GLK2-R1	ATGTCGATGGGAGGATTAGTGGGT
GFP	qRT-GFP-F1	CTGCCCGACAACCACTACCT
	qRT-GFP-R1	GCTCGTCCATGCCGTGAG
nYFP	qRT-nYFP-F1	GACGTAAACGGCCACAAGTT
	qRT-nYFP-R1	CGTAGCCGAAGGTGGTCAC
cYFP	qRT-cYFP-F1	GCTGCTGCCCGACAACC
	qRT-cYFP-R1	GTCCATGCCGAGAGTGATCC
BGH2	qRT-BGH2-F1	GGAAAGAGCTAGCGAAAGAG
	qRT-BGH2-R1	GAAAGCAAATCACGGATTCT
BGH3	qRT-BGH3-F1	ACATTCTTACATTCGCATGC
	qRT-BGH3-R1	GGTGGTCTAACGCCTTGAC

Supplementary Table S6: Primers list for production of recombinant protein

Primer name	Sequence (5' - 3')
In-Fusion-pMAL-c4X-F	GACTGGGAAAACCCTGGCGTTA
In-Fusion-pMAL-c4X-R	CAAGCTTGCCTGCAGGTCGAC
BPG4-In-Fusion-pMAL-c4X-F	CTGCAGGCAAGCTTGATGGCAGATTCTTCTTCTGCT
BPG4-In-Fusion-pMAL-c4X-R	AGGGTTTTCCCAGTCTCAGTCGTCGGATTCTTTAGA
In-Fusion-pGEX-6P-3-F	GACTGACGATCTGCCTCGC
In-Fusion-pGEX-6P-3-R	GGGCCCCTGGAACAGAAC
GLK1-In-Fusion-pGEX-6P-3-F	CTGTTCCAGGGGCCCATGTTAGCTCTGTCTCCGGC
GLK1-In-Fusion-pGEX-6P-3-R	GGCAGATCGTCAGTCTCAGGCACAAGACGCGGTCG

Supplementary Table S7: Primers list for ChIP-qPCR

Primer name	Sequence (5' - 3')
ChIP-BPG4-promoter-F1	ATGTATAGAGAGAGAGGGGCC
ChIP-BPG4-promoter-R1	ATATGCATGCATGGGGCCAGA
ChIP-BPG4-exon-F1	GGAATTCTTCAAGGCGTATGA
ChIP-BPG4-exon-R1	CAGTCGTCGGATTCTTTAGATG
ChIP-BPG4-intron-F1	CTACAGATAGGATCTAGCGTGATG
ChIP-BPG4-intron-R1	TCTAGTCGCCAGGTAGTCAAGT

Supplementary Table S8: Primers list for protoplast transient expression assays

Primer name	Sequence (5' - 3')
pGLHNew_RLH-In-Fusion-F1	GAGCTCGGCGCGCCTTAATTAA
pGLHNew_RLH-In-Fusion-R1	GGGAATTCGTACCGGCCAGTTA
HEMA1-In-fusion-pGLHNew_RLH-F1	CCGGTACGAATTCCCggtcaattggttagcatcca
HEMA1-In-fusion-pGLHNew_RLH-R1	AGGCGCGCCGAGCTCtgaaacccaaaatctcaaatctc
GUN4-In-fusion-pGLHNew_RLH-F1	CCGGTACGAATTCCCagacgcatttacatgcacaa
GUN4-In-fusion-pGLHNew_RLH-R1	AGGCGCGCCGAGCTCtgatggagaggaagagagag
CAO-In-fusion-pGLHNew_RLH-F1	CCGGTACGAATTCCCaaacgaaataacttttagatttagt
CAO-In-fusion-pGLHNew_RLH-R1	AGGCGCGCCGAGCTCggcgtcaccggaaagaga
BPG4-In-fusion-pGLHNew_RLH-F1	CCGGTACGAATTCCCccggtatactatacgtatatgattatctctaagccaaa
BPG4-In-fusion-pGLHNew_RLH-R1	AGGCGCGCCGAGCTCtgttaatggaaaccctagaattaacacccaa
BPG4pro-G-box-1-mutation-F1	tggatAAAAAAgaggatgcgtgagatggaaatga
BPG4pro-G-box-1-mutation-R1	tcctcTTTTTTatccatggcccctctctctctat
BPG4pro-G-box-2-mutation-F1	tctccAAAAAAgggcttctggccccatgcatgca
BPG4pro-G-box-2-mutation-R1	agcccTTTTTTggagattgatcataattggctga

Supplementary Table S9: Primers list for EMSA

Primer name	Sequence (5' - 3')
BPG4-promoter-G-box-1-F	GGGGCCATGGATCACGTGGAGGATGCGTGA
BPG4-promoter-G-box-1-R	TCACGCATCCTCCACGTGATCCATGGCCCC
BPG4-promoter-G-box-2-F	TGATCAATCTCCCACGTGGGGCTTCTGGCC
BPG4-promoter-G-box-2-R	GGCCAGAAGCCCCACGTGGGAGATTGATCA
BPG4-G-box-1-mutation-F	GGGGCCATGGATAAAAAAGAGGATGCGTGA
BPG4-G-box-1-mutation-R	TCACGCATCCTCTTTTTTATCCATGGCCCC
BPG4-G-box-2-mutation-F	TGATCAATCTCCAAAAAAGGGCTTCTGGCC
BPG4-G-box-2-mutation-R	GGCCAGAAGCCCTTTTTTGGAGATTGATCA
CAO-probe-F	TTTAGATCCCATGATTCTCAACACGACTGA
CAO-probe-R	TCAGTCGTGTTGAGAATCATGGGATCTAAA

Supplementary Table S10: Primers list for Y2H, BiFC, and CoIP

Primer name	Sequence (5' - 3')
BPG4-CDS-F1	CACCATGGCAGATTCTTCTTCTGCT
BPG4-CDS-R1	TCAGTCGTCGGATTCTTTAGA
GLK1-CDS-F1	CACCATGTTAGCTCTGTCTCCGGC
GLK1-CDS-R1	TCAGGCACAAGACGCGGTC
GLK2-CDS-F1	CACCATGTTAACTGTTTCTCCGGCT
GLK2-CDS-R1	TCAAGGAAGAGGAGGAACATT
HY5-CDS-F1	CACCATGCAGGAACAAGCGACTAG
HY5-CDS-R1	TCAAAGGCTTGCATCAGCATT
COP1-CDS-F1	CACCATGGAAGAGATTTCGACGGAT
COP1-CDS-R1	TCACGCAGCGAGTACCAGA
BIN2-CDS-F1	CACCATGGCTGATGATAAGGAGAT
BIN2-CDS-R1	TTAAGTTCCAGATTGATTCA
GLK1-N-terminal-F1	caccATGTTAGCTCTGTCTCCGGC
GLK1-N-terminal-R1	tcaACTGATCCGATTGTTCTTGGA
GLK1-DBD-F1	caccAACAACGAAGGGAAGAGAAAG
GLK1-DBD-R1	tcaATGTTTCCGATGAGACCTATAT
GLK1-PRD-F1	caccTTGCTAGCTCGTGAGGCCG
GLK1-PRD-R1	tcaCGGATGTAAGTCTACCGGAG
GLK1-GCT-box-F1	caccTCAAAAGAGAGCGTGGATGC
GLK1-GCT-box-R1	TCAGGCACAAGACGCGGTC
BPG4-A_thal_3526-F1	caccATGGCAGATTCTTCTTCTGCT
BPG4-A_thal_3526-R1	tcaCCTCTCCTCATACGCCTTG
BPG4-C-terminal-F1	caccCAAAGCAAACAAGAGCAAATGT
BPG4-C-terminal-R1	TCAGTCGTCGGATTCTTTAGA
