# Supplementary material for: BPG4 regulates chloroplast development and homeostasis by suppressing GLK transcription factors and involving light and brassinosteroid signaling
Source: Nat Commun. 2024 Jan 8;15:370. doi: 10.1038/s41467-023-44492-5 (PMC10774444; doi:10.1038/s41467-023-44492-5)
Supplement: Supplementary file 3 — Reporting Summary [file 41467_2023_44492_MOESM3_ESM.pdf]

nature portfolio

Corresponding author(s): Tekeshi Nakano

Last updated by author(s): Dec 5, 2023

Reporting Summary

Nature Portfolio wishes to improve the reproducibility of the work that we publish. This form provides structure for consistency and transparency in reporting. For further information on Nature Portfolio policies, see our Editorial Policies and the Editorial Policy Checklist.

Statistics

For all statistical analyses, confirm that the following items are present in the figure legend, table legend, main text, or Methods section.

n/a	Confirmed	
☐	☒	The exact sample size (*n*) for each experimental group/condition, given as a discrete number and unit of measurement
☐	☒	A statement on whether measurements were taken from distinct samples or whether the same sample was measured repeatedly
☐	☒	The statistical test(s) used AND whether they are one- or two-sided<br>*Only common tests should be described solely by name; describe more complex techniques in the Methods section.*
☒	☐	A description of all covariates tested
☐	☒	A description of any assumptions or corrections, such as tests of normality and adjustment for multiple comparisons
☐	☒	A full description of the statistical parameters including central tendency (e.g. means) or other basic estimates (e.g. regression coefficient) AND variation (e.g. standard deviation) or associated estimates of uncertainty (e.g. confidence intervals)
☐	☒	For null hypothesis testing, the test statistic (e.g. *F*, *t*, *r*) with confidence intervals, effect sizes, degrees of freedom and *P* value noted<br>*Give P values as exact values whenever suitable.*
☒	☐	For Bayesian analysis, information on the choice of priors and Markov chain Monte Carlo settings
☒	☐	For hierarchical and complex designs, identification of the appropriate level for tests and full reporting of outcomes
☒	☐	Estimates of effect sizes (e.g. Cohen's *d*, Pearson's *r*), indicating how they were calculated

Our web collection on statistics for biologists contains articles on many of the points above.

Software and code

Policy information about availability of computer code

Data collection	Thermal Cyclar Dice Real Time System (takara) was used to quantitatively measure mRNA level.<br>LAS-4000 mini (fujifilm) was used for chemiluminescence detection during western blot.<br>ChemiDoc TouchTM Imaging System (BIO-RAD) was used for EMSAs.<br>SpectraMax i3x and SpectraMax iD5 (MOLECULAR DEVICES) was used to detect luciferase signals.<br>LSM700 microscope (Zeiss) was used for Confocal laser scanning microscopy.<br>FluorCam 800MF (Photon Systems Instruments) was used for measuring the Fv/Fm value.
Data analysis	Microsoft Excel (Microsoft), R environment, Photoshop Elements 2022 (Adobe), iTOL v6 (https://itol.embl.de/)

For manuscripts utilizing custom algorithms or software that are central to the research but not yet described in published literature, software must be made available to editors and reviewers. We strongly encourage code deposition in a community repository (e.g. GitHub). See the Nature Portfolio guidelines for submitting code & software for further information.

April 2023

Data

Policy information about availability of data

All manuscripts must include a data availability statement. This statement should provide the following information, where applicable:

- Accession codes, unique identifiers, or web links for publicly available datasets
- A description of any restrictions on data availability
- For clinical datasets or third party data, please ensure that the statement adheres to our policy

Arabidopsis mutants used in the current study are available from the corresponding author. All the unprocessed data, gels, and blots were available within the paper and its Supplementary Information, and provided in the Source Data file. Source data are provided in this paper.

Research involving human participants, their data, or biological material

Policy information about studies with human participants or human data. See also policy information about sex, gender (identity/presentation), and sexual orientation and race, ethnicity and racism.

Reporting on sex and gender	n/a
Reporting on race, ethnicity, or other socially relevant groupings	n/a
Population characteristics	n/a
Recruitment	n/a
Ethics oversight	n/a

Note that full information on the approval of the study protocol must also be provided in the manuscript.

Field-specific reporting

Please select the one below that is the best fit for your research. If you are not sure, read the appropriate sections before making your selection.

☒ Life sciences ☐ Behavioural & social sciences ☐ Ecological, evolutionary & environmental sciences

For a reference copy of the document with all sections, see nature.com/documents/nr-reporting-summary-flat.pdf

Life sciences study design

All studies must disclose on these points even when the disclosure is negative.

Sample size	No sample-size calculation was performed. We set sample sizes based on our preliminary data and published papers by other researchers.
Data exclusions	No data were excluded from analysis.
Replication	All phenotype analysis, qRT–PCR analysis, observation for confocal laser scanning microscopy images, Y2H, BiFC, EMSAs, transient assay, GUS staining, visualization of ROS, and measurement of photosynthetic activity were repeated at least twice independently with similar results.
Randomization	Plant materials were randomly selected from a larger pool of plants.
Blinding	Investigators were not blinded to group allocation during data collection and/or analysis because there is no group allocation involved in this study.

Reporting for specific materials, systems and methods

We require information from authors about some types of materials, experimental systems and methods used in many studies. Here, indicate whether each material, system or method listed is relevant to your study. If you are not sure if a list item applies to your research, read the appropriate section before selecting a response.

Materials & experimental systems

n/a	Involved in the study
☐	☒ Antibodies
☒	☐ Eukaryotic cell lines
☒	☐ Palaeontology and archaeology
☒	☐ Animals and other organisms
☒	☐ Clinical data
☒	☐ Dual use research of concern
☐	☒ Plants

Methods

n/a	Involved in the study
☒	☐ ChIP-seq
☒	☐ Flow cytometry
☒	☐ MRI-based neuroimaging

Antibodies

Antibodies used	A rabbit polyclonal antibody against BPG4 (anti-BPG4 antibody) was generated in this study, using the MBP-BPG4 recombinant protein.<br>Anti-ACTIN antibody (0869100-CF, MP Biomedical)<br>Anti-Myc antibody (9E10, Sigma Aldrich)<br>Anti-GFP antibody (A11122, Molecular Probes)<br>Anti-rabbit horseradish peroxidase-conjugated secondary antibody (W4018, Promega)<br>Anti-mouse horseradish peroxidase-conjugated secondary antibody (W4028, Promega)<br>normal rabbit IgG (Dako, Glostrup, Denmark, #X0936)<br>Donkey anti-rabbit IgG secondary antibody, Alexa Fluor™ Plus 647 (Invitrogen #A32795, 1:200 dilution)
Validation	The performance of the generated anti-BPG4 antibody was confirmed by the use of Col-0, bpg4-1, and BPG4-OX-2. Anti-BPG4 antibody was used at a 1:30,000 dilution together with anti-rabbit horseradish peroxidase-conjugated secondary antibody (1:90,000).<br>Anti-ACTIN antibody was used at a 1:20,000 dilution together with anti-mouse horseradish peroxidase-conjugated secondary antibody (1:60,000).<br>Anti-Myc antibody was used at a 1:5,000 dilution together with anti-mouse horseradish peroxidase-conjugated secondary antibody (1:10,000).<br>Anti-GFP antibody was used at a 1:20,000 dilution together with anti-rabbit horseradish peroxidase-conjugated secondary antibody (1:50,000 or 1:40,000).

Dual use research of concern

Policy information about dual use research of concern

Hazards

Could the accidental, deliberate or reckless misuse of agents or technologies generated in the work, or the application of information presented in the manuscript, pose a threat to:

No	Yes
☒	☐ Public health
☒	☐ National security
☒	☐ Crops and/or livestock
☒	☐ Ecosystems
☒	☐ Any other significant area

Experiments of concern

Does the work involve any of these experiments of concern:

No	Yes
☒	☐ Demonstrate how to render a vaccine ineffective
☒	☐ Confer resistance to therapeutically useful antibiotics or antiviral agents
☒	☐ Enhance the virulence of a pathogen or render a nonpathogen virulent
☒	☐ Increase transmissibility of a pathogen
☒	☐ Alter the host range of a pathogen
☒	☐ Enable evasion of diagnostic/detection modalities
☒	☐ Enable the weaponization of a biological agent or toxin
☒	☐ Any other potentially harmful combination of experiments and agents

Plants

Seed stocks	Arabidopsis ecotypes Columbia (Col-0) and Wassilewskija (Ws) were used as WT plants. bpg4-1 (CS927130), bpg4-2 (CS391584), 35S:GLK1-GFP/glk1glk2 (CS2107720), and 35S:GLK2-GFP (CS71752) were obtained from the ABRC.<br>det2, bri1-5, bin2-1, bes1-D, gsk3 quadruple, BRI1-OX and glk1glk2 were described previously9,25,74–78.
Novel plant genotypes	bpg4-1D was screened in FOX line.<br>bpg4-1 and bpg4-2 were isolated in ABRC T-DNA insertion lines.<br>bgh1-1, bgh2-1, bgh2-2, bgh3-1, and bgh3-2 mutants were generated using the CRISPR–Cas9 system.<br>Two independently genotyped BPG4-OX, BGH2-OX, BGH3-OX were generated using Agrobacteirum by floral dipping methods.<br>35S:BPG4-GFP and BPG4pro:GUS was generated in the same way.<br>bpg4glk1glk2 and BPG4-OXGLK1-OX were generated by crossing.
Authentication	Transgenic plants were selected by resistance to antibiotics.<br>Genome-edited plants were selected by sequencing.<br>T-DNA inserted lines were selected by PCR genotyping.
